# Interferon-Inducible IFI16, a Negative Regulator of Cell Growth, Down-Regulates Expression of Human Telomerase Reverse Transcriptase (*hTERT*) Gene

**DOI:** 10.1371/journal.pone.0008569

**Published:** 2010-01-05

**Authors:** Lynda Li Song, Larissa Ponomareva, Hui Shen, Xin Duan, Fatouma Alimirah, Divaker Choubey

**Affiliations:** 1 Department of Environmental Health, Cincinnati, Ohio, United States of America; 2 Cincinnati VA Medical Center, Cincinnati, Ohio, United States of America; 3 Hines VA Hospital, Hines, Illinois, United States of America; Roswell Park Cancer Institute, United States of America

## Abstract

**Background:**

Increased levels of interferon (IFN)-inducible IFI16 protein (encoded by the *IFI16* gene located at 1q22) in human normal prostate epithelial cells and diploid fibroblasts (HDFs) are associated with the onset of cellular senescence. However, the molecular mechanisms by which the IFI16 protein contributes to cellular senescence-associated cell growth arrest remain to be elucidated. Here, we report that increased levels of IFI16 protein in normal HDFs and in HeLa cells negatively regulate the expression of human telomerase reverse transcriptase (*hTERT*) gene.

**Methodology/Principal Findings:**

We optimized conditions for real-time PCR, immunoblotting, and telomere repeat amplification protocol (TRAP) assays to detect relatively low levels of hTERT mRNA, protein, and telomerase activity that are found in HDFs. Using the optimized conditions, we report that treatment of HDFs with inhibitors of cell cycle progression, such as aphidicolin or CGK1026, which resulted in reduced steady-state levels of IFI16 mRNA and protein, was associated with increases in hTERT mRNA and protein levels and telomerase activity. In contrast, knockdown of IFI16 expression in cells increased the expression of c-Myc, a positive regulator of *hTERT* expression. Additionally, over-expression of IFI16 protein in cells inhibited the c-Myc-mediated stimulation of the activity of hTERT-luc-reporter and reduced the steady-state levels of c-Myc and hTERT.

**Conclusions/Significance:**

These data demonstrated that increased levels of IFI16 protein in HDFs down-regulate the expression of *hTERT* gene. Our observations will serve basis to understand how increased cellular levels of the IFI16 protein may contribute to certain aging-dependent diseases.

## Introduction

The interferon (IFN) family of cytokines exhibits multiple biological activities both *in vitro* and *in vivo* [1]–[4]. The family includes type-I (IFN-α and IFN-β) and type-II (IFN-γ) IFNs among others [1]–[3]. The biological activities of IFNs include the cell growth-inhibitory activities [1]–[5]. Studies have suggested that expression of a set of IFN-inducible genes, which encode proteins that mediate the biological activities of IFNs [1], [6], is up-regulated during the onset of cellular senescence in a variety of human cells [7]–[11]. Moreover, the loss of expression of IFN-inducible genes is correlated with immortalization of cells and the development of certain human cancers [7], [11]. These observations have suggested a role for IFN-inducible proteins in the regulation of cellular senescence.

Our studies [12]–[14] have revealed that increased expression of IFN-inducible IFI16 protein in human normal prostate epithelial cells and HDFs contributes to cellular senescence. These studies demonstrated that knockdown of IFI16 expression in HDFs prolonged the proliferation potential [12], whereas overexpression of IFI16 protein in PC-3 human prostate cancer cell line resulted in senescence-like phenotype and reduced telomere length [13]. The IFI16 protein is a member of structurally and functionally-related family of proteins (the p200-proteins) [14]. The family includes the murine (for example, p202a p202b, p203, and p204 etc.) and human (for example, IFI16, MNDA, IFIX, and AIM2) proteins. Increased expression of some of the p200-family of proteins inhibits cell cycle progression by inhibiting the transcriptional activities of a variety of growth-promoting transcription factors [15]–[17]. For example, increased levels of the p202 protein (encoded by the *Ifi202a* and *Ifi202b* genes) inhibit c-Myc-mediated transcription [18]. Additionally, the p202 protein binds to the pRb pocket and E2Fs (E2F1 and E2F4) and inhibits the E2F-stimulated transcription of growth-promoting genes [19]–[21]. Similarly, the IFI16 protein can also bind to pRb protein and increased levels of IFI16 protein in prostate cancer cells inhibit the E2F1-mediated transcription [13], [14]. Additionally, overexpression of IFI16 protein in human osteosarcoma cell line Saos-2 down-regulated the expression of c-*MYC* and *RAS* genes [22]. Moreover, the IFI16 protein can bind to the promoter of the *c-MYC* gene in chromatin immunoprecipitation assays [23]. Although, these observations suggest that increased levels of IFI16 protein negative regulate the expression of *c-MYC* in certain tumor cell lines, it remains unknown how increased levels of the IFI16 protein in human normal cells contribute to cellular senescence-associated cell growth arrest.

The telomere length is believed to be an important determinant of cellular longevity and immortal cells often employ telomerase, a ribonucleoprotein that elongates telomeres, to maintain telomere length [24]–[26]. Indeed, increased expression of the catalytic subunit of human telomerase reverse transcriptase (hTERT) results in immortalization of certain human primary fibroblasts and epithelial cells [24], [25].

Most somatic cells are reported to express low levels of hTERT protein [27]–[30] and disruption of the activity in normal cells slows cell proliferation, restricts cell lifespan, and alters the maintenance of the 3′-single stranded telomeric overhang without changing the rate of overall telomere shortening [25], [26]. However, most tumor cells possess relatively high telomerase activity [28], [29]. This differential display of telomerase activity is largely attributed to the ability of tumor cells to up-regulate the expression of *hTERT* gene [24].

Several cell signaling pathways regulate the activity of transcription factors and co-regulators that regulate the expression of *hTERT* gene [31], [32]. The pathways that negatively regulate the expression of *hTERT* include the IFN-signaling pathway [33]–[37]. It is known that IFN-treatment of certain cells down-regulates *hTERT* expression and inhibits telomerase activity [33], [35], [37]. Additionally, the pRb/E2F pathway negatively regulates *hTERT* expression [29], [38]. It has been shown that the E2F-pocket protein-histone deacetylase transcriptional repressor complex serves as a key mechanistic basis for the repression of the *hTERT* gene in normal human cells during the G_1_ phase of cell cycle [29]. Activation of cell signaling pathways by growth factors that results in up-regulation of c-Myc expression positively regulate the expression of *hTERT* gene [39]–[41].

The c-Myc oncoprotein is a transcription factor with basic, helix-loop-helix, and leucine zipper domains (bHLHLZ) [42]. High-affinity sequence-specific DNA-binding of c-Myc requires the heterodimeric partner Max [42]. A number of genes have been implicated as the transcriptional targets of c-Myc, including the *hTERT* gene [39]–[41]. Expression of c-Myc is down-regulated by *in vitro* treatment of certain human cells and cell lines with IFNs [43], [44].

Our previous observations that increased levels of IFI16 protein in human normal prostate epithelial cells [13] and fibroblasts [12] are associated with cellular senescence-associated cell growth arrest and immortalization of HDFs with hTERT results in down-regulation of IFI16 expression prompted us to test whether IFI16 protein could regulate the expression of *hTERT*. Our observations revealed that increased levels of IFI16 protein down-regulate the expression of *hTERT*, in part, through inhibiting the c-Myc-mediated transcription of the *hTERT* gene.

## Materials and Methods

### Cell Lines, Culture Conditions, and Treatments

HeLa cells were generously provided by Dr. Olivia Perriera-Smith (University of Texas Health Science Center at San Antonio, TX). Normal human fetal lung fibroblasts (AG06814N, WI-38) at population doubling 15 (passage 12) were obtained from the National Institute of Aging Cell Culture Repository (Coriell Medical for Medical Research, Camden, NJ). HeLa cells and WI-38 cell cultures were maintained in DMEM culture media with high glucose, which was supplemented with 10% fetal bovine serum and antibiotics (Invitrogen). Sub-confluent cultures of cells, when indicated, were treated with either aphidicolin (5 µg/ml, Calbiochem, San Diego, CA) or CGK1026 (10 µM) for the indicated duration.

### Plasmids and Expression Vectors

The plasmid pCMV-IFI16 has been described previously [13]. The plasmid (pCMV-c-Myc) encoding the human c-Myc protein was originally provided by Dr. Robert Eisenman (Fred Hutchinson Cancer Research Center, Seattle, WA). The pMyc-TA-luc reporter plasmid was purchased from BD Biosciences Clontech (Palo Alto, CA) as a part of the BD Bioscience Pathway Profiling System. The reporter contains six tandem copies of the E-box (c-Myc DNA-binding sequence) consensus sequence, a minimal TA promoter (the TATA box from the herpes simplex virus thymidine kinase promoter), and the downstream to the minimal promoter the firefly luciferase reporter gene. The hTERT-luc-reporter plasmid containing the 5′-regulatory region (from −1125 bp to −43 bp) of the human *hTERT* gene has been described previously [45].

### Overexpression or Knockdown of IFI16 Expression

Sub-confluent cultures of HeLa cells were either infected with retroviral vector (LZRS-IFI16 or LZRS-IFI16AS) that allowed expression of mRNA encoding the IFI16 protein and an antisense to IFI16 mRNA, respectively. As a control, we infected cells with a retroviral LZRS vector with only a linker sequence. To over-express IFI16 protein in WI-38 cells, cells were nucleofected with pCMV-IFI16 plasmid. As a negative control, cells were nucleofected with an empty pCMV vector. To knockdown expression of IFI16 in WI-38 cells, we used a pool of IFI16 siRNAs (purchased from Dharmacon, Denver, CO) or a nonspecific control siRNA (cat # D-001206-02-05) as recommended by the manufacturer using Lipofectamine (Invitrogen) transfection agent. 60 h post-transfections, cells were processed for immunoblotting.

### Nucleofections

WI-38 cells were nucleofected with 2 µg of pCMV-IFI16 plasmid or pCMV highly purified (endotoxin-free) plasmid. The Nucleofector-II device (Amaxa Biosystems, Germany) was used (Nucleofection kit R and program V-001) as suggested by the supplier. After nucleofections, cells were harvested at the indicated times to isolate total RNA or to prepare total cell extracts.

### Telomere Repeat Amplification Protocol Assays

To detect relatively low levels of the activity of telomerase in human diploid fibroblasts, we optimized the assay conditions using nuclear extracts prepared from decreasing numbers (2000, 1000, 500, 250, and 125) of HeLa cells that are known to express the detectable levels of telomerase [46]. The telomerase activity was detected using a TRAPeze Telomerase activity detection kit (Chemicon International, Temecula, CA) as suggested by the supplier.

### Immunoblotting and Antibodies

To detect low basal levels of hTERT protein in human normal WI-38 and BJ fibroblasts, we used nuclear extracts and our optimized immunoblotting conditions to detect high molecular weight proteins [47]. In brief, after fractionation of cells into the cytoplasmic and nuclear fractions, the nuclear fraction was incubated with RIPA buffer and the lysates were subjected to SDS-PAGE electrophoresis using pre-casted gels from Nupage Bis-Tris (4–12%) gel systems (Invitrogen). Proteins were transferred to Immobilon-P (Millipore, Billerica, MA) membranes and immunoblotting was performed as described previously [47]. Antibodies specific for IFI16 (sc-8023) and c-Myc (sc-40) were purchased from Santa Cruz Biotech (Santa Cruz, CA). To detect human hTERT protein, we used either a mouse monoclonal antibody (NB100–317; from Novus Biologicals, Littleton, CO) or a rabbit polyclonal antibody (sc-7212; from Santa Cruz Biotech, CA). Antibody (cat # 4967) to β-actin was purchased from Cell Signaling Technology (Danvers, MA). Horseradish peroxidase (HRP) conjugated secondary anti-mouse (NXA-931) and anti-rabbit (NA-934) antibodies were from Amersham Biosciences.

### Reverse Transcriptase Reaction and Real-Time PCR

Total RNA was isolated from WI-38 fibroblasts with Trizol reagent (Invitrogen, Carlsbad, CA, USA). cDNA synthesis was done using primers with SuperScript First-strand Synthesis System for RT-PCR (Invitrogen, Carlsbad, CA, USA). Quantitative real-time TaqMan PCR technology (Applied Biosystems, Foster City, CA, USA) was used. The PCR cycling program consisted of denaturing at 95°C for 10 min and 40 cycles at 95°C for 15 seconds, and annealing and elongation at 60°C for 1 min. The TaqMan assays for *IFI16* (assay Id #Hs00194216_m1), *hTERT* (assay Id# Hs00972646_m1), and for the endogenous control β-actin (assay Id# Hs99999903_ml) were purchased from Applied Biosystems (Foster City, CA) and used as suggested by the supplier.

### Reporter Assays

All transient transfection assays were performed using FuGene6 transfection reagent (Roche, Indianapolis, IN) according to the manufacturer's instructions. In brief, sub-confluent cells were co-transfected with desired reporter plasmid (c-Myc-luc or hTERT-luc; 1.8 µg) along with pRL-TK plasmid (0.2 µg) as an internal control. 48 h after transfections of cells, firefly luciferase and Renilla luciferase activities were assayed using dual-luciferase reporter assay kit (Promega, Madison, WI). Relative luciferase activity was expressed as the ratio of the firefly luciferase and *Renilla* luciferase activities. Student's *t-*test for paired samples was used to determine statistical significance of the reporter activity data. Differences were considered statistically significant at *P*≤0.05.

## Results

### Expression of IFI16 Is Inversely Correlated with the hTERT Expression

**Figure 1:**
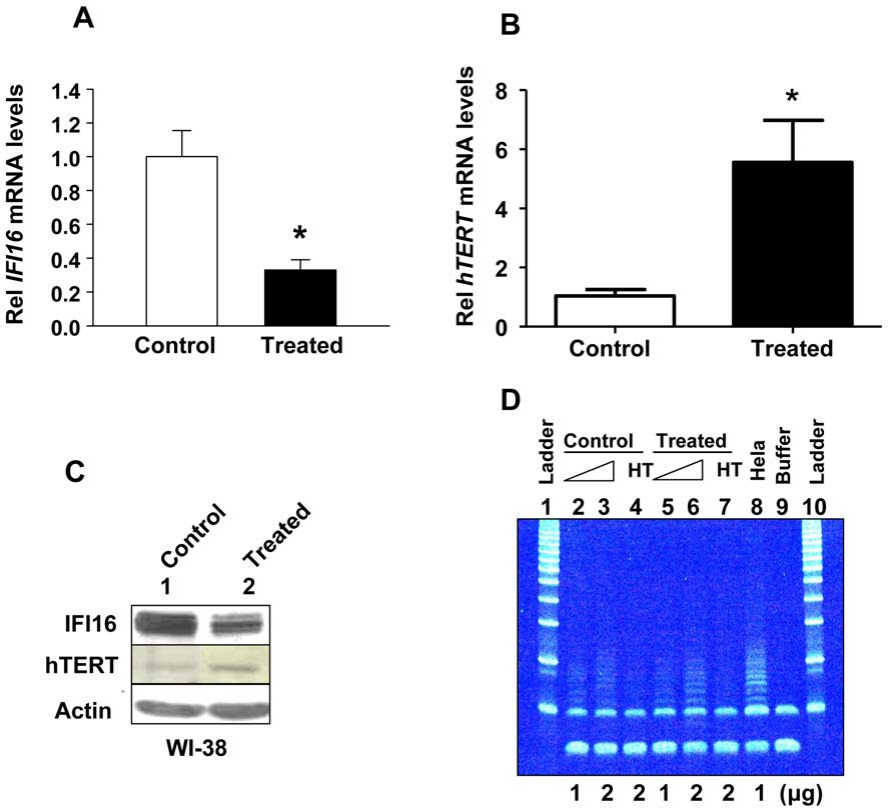
Reduced expression levels of IFI16 protein in aphidicolin-treated human normal diploid fibroblasts are associated with increased expression levels of the hTERT and telomerase activity. (**A** and **B**) Total RNA isolated from untreated (control) or aphidicolin (5 µg/ml for 24 h) treated young WI-38 fibroblasts was subjected cDNA synthesis followed by quantitative real-time PCR using the TaqMan assay for the *IFI16* gene (A) or *hTERT* gene (B). Results are mean values of triplicate experiments and the error bars represent standard deviation (^*^ *p<0.05*). (**C**) Total protein extracts prepared from untreated (lane 1) or aphidicolin (5 µg/ml for 24 h) treated (lane 2) young WI-38 fibroblasts were subjected immunoblotting using antibodies specific to the indicated proteins. (**D**) Extracts containing the indicated amounts (µg) of proteins from control (lanes 2–4) or aphidicolin-treated (lanes 5–7) young WI-38 cells were subjected to TRAPeze assays without any treatment (lanes 2, 3, 5, and 6) or after heat treatment (lanes 4 and 7) to detect the telomerase activity. As controls, extracts from HeLa cells (lane 8) or buffer alone (lane 9) were also included in the assay. The reaction products were subjected to native polyacrylamide gel electrophoresis along with DNA fragments of increasing length as size markers (DNA-ladder).

Our earlier observations [12] that immortalization of human diploid fibroblasts with SV40 large T antigen or hTERT reduced steady-state levels of IFI16 mRNA and protein prompted us to test whether increased levels of IFI16 protein in human diploid fibroblasts could regulate the *hTERT* expression. Because expression of *hTERT* gene in normal HDFs is up-regulated transiently in the S-phase of cell cycle [28], we chose to treat young WI-38 HDFs with aphidicolin (a known inhibitor of DNA synthesis that accumulates cells in the early S phase of the cell cycle; ref. 48), to examine correlation between IFI16 and hTERT expression. Treatment of HDFs with aphidicolin at a concentration (5 µg/ml), which has been used previously [28], for 24 h did not result in any measurable toxicity. Notably, the treatment of cells with aphidicolin resulted in significant decreases in the steady-state levels of IFI16 mRNA (Fig. 1A) and protein (Fig. 1C). Interestingly, using our optimized conditions to specifically detect low levels of hTERT mRNA and protein, we found that decreases in the steady-state levels of IFI16 mRNA and protein were inversely correlated with the hTERT mRNA (Fig. 1B) and protein (Fig. 1C) levels. Consistent with these observations, we also noted moderate increases in the activity of telomerase after the treatment of cells (Fig. 1D; compare the intensity of ladder in lane 3 with lane 6).

**Figure 2:**
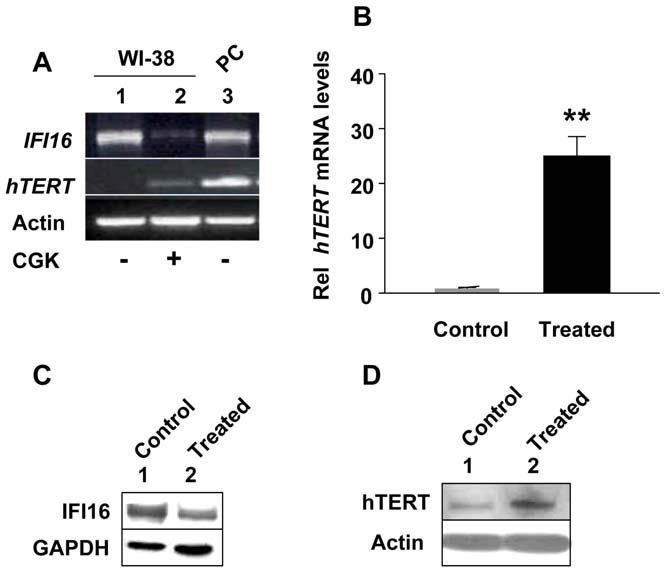
Reduced expression levels of IFI16 protein in human normal diploid fibroblasts after treatment with histone deacetylase inhibitor are associated with increased expression of hTERT and increased telomerase activity. (**A**) Total RNA isolated from untreated (control, lane 1) or CGK1026 (10 µM for 24 h, lane 2) treated young WI-38 fibroblasts was subjected cDNA synthesis followed by semi-quantitative PCR using a pair of primer specific to the *IFI16*, *hTERT*, or actin. As a positive control, we used RNA from human HT1080, a human fibrosarcoma cell line. (**B**) Total RNA isolated from untreated (control) or CGK1026 (10 µM for 24 h; treated) treated young WI-38 fibroblasts was subjected cDNA synthesis, followed by quantitative real-time PCR using the TaqMan assay for the *hTERT* gene. Results are mean values of triplicate experiments and error bars represent standard deviation (^**^ *p<0.005*). (**C** and **D**) Total protein extracts prepared from untreated (lane 1) or CGK1026 (10 µM for 24 h; treated) treated young WI-38 fibroblasts were subjected to immunoblotting using antibodies specific to the indicated proteins.

Inhibition of histone deacetylase (HDAC) activity in certain cells inhibits interferon signaling and expression of IFN-inducible genes [49]. Therefore, we tested whether treatment of WI-38 fibroblasts with an inhibitor of HDAC (CGK1026), which was reported [29] to activate transcription of *hTERT* gene by inhibiting the activity of the Rb-E2F transcriptional repressor complex, has any effect on IFI16 and hTERT expression. Treatment of WI-38 cells with CGK1026 at a concentration (10 µM), which has been used previously [49], for 24 h did not result in any detectable morphological changes and resulted in significant decreases in the steady-state basal levels of IFI16 mRNA (Fig. 2A). Interestingly, the decreases in IFI16 mRNA levels were associated with increases in hTERT mRNA levels as determined using the optimized conditions for semi-quantitative RT-PCR (Fig. 2A) and quantitative real-time PCR (Fig. 2B) to detect low basal levels of hTERT mRNA. Furthermore, this inverse correlation between IFI16 and hTERT mRNA levels was also seen at the protein levels (Fig. 2C and 2D). Notably, basal low levels of hTERT mRNA were not detectable in unsynchronized cultures of WI-38 cells (Fig. 2A, compare lane 2 with 1). However, basal levels of hTERT protein were detectable in unsynchronized cultures of WI-38 cells (Fig. 2D, compare lane 2 with 1). This could be due to a relatively short half-life (∼2 h) of the hTERT mRNA [50] as compared to the protein [51]. Taken together, our above observations clearly demonstrated that the expression of IFI16 protein is inversely correlated with the hTERT expression in aphidicolin or CGK1026 treated WI-38 HDFs.

### Increased Levels of IFI16 Protein in HeLa Cells Are Associated with Reduced Levels of c-Myc and hTERT

**Figure 3:**
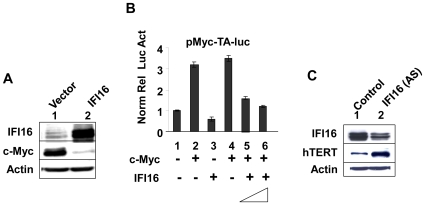
IFI16 inhibits c-Myc-stimulated transcription and hTERT expression in HeLa cells. (**A**) Total protein extracts prepared from HeLa cells infected with control retrovirus (lane 1) or a virus encoding IFI16 protein (lane 2) were subjected to immunoblotting using antibodies specific to the indicated proteins. (**B**) Sub-confluent cultures of HeLa cells were transfected with pMyc-TA-luc reporter plasmid (1.0 µg) along with a second pRL-TK reporter plasmid (0.2 µg) and an empty plasmid (pCMV; column 1), a plasmid encoding c-Myc (column 2 and 4), a plasmid encoding IFI16 (column 3), or both plasmids encoding c-Myc and increasing amounts of the plasmid encoding IFI16 protein (column 5 and 6). After 44–48 h of transfections, cells were lysed and the lysates were analyzed for dual luciferase activity. Normalized relative luciferase activity in control cells is indicated as 1.0. (**C**) Total protein extracts prepared from HeLa cells infected with control retrovirus (lane 1) or a virus encoding antisense to IFI16 mRNA (lanes 2) were subjected to immunoblotting using antibodies specific to the indicated proteins.

Forced overexpression of IFI16 protein in Saos2 (a human osteosarcoma cell line) cells down-regulated the expression of *c-MYC* gene [22]. Furthermore, IFI16 protein can bind to the promoter of the *c-MYC* gene [23]. Because the promoter region of the *hTERT* gene contains c-Myc-responsive elements (E-boxes) and the transcription of *hTERT* gene is activated by c-Myc [39]–[41], we explored whether increased levels of IFI16 protein in cells could negatively regulate the expression of *hTERT* gene through inhibition of c-Myc-stimulated transcription. Because expression of c-Myc, hTERT, and IFI16 is readily detectable in HeLa cells and IFN-treatment of HeLa cells results in down-regulation of c-Myc [52], we chose these cells to investigate the molecular mechanisms by which IFN-inducible IFI16 protein may regulate the *hTERT* expression. As shown in Fig. 3A, overexpression of IFI16 protein in HeLa cells significantly decreased the steady-state levels of c-Myc protein. This observation prompted us to test whether increased levels of IFI16 protein inhibit c-Myc-stimulated transcription. As shown in Fig. 3B, transfection of a plasmid encoding c-Myc protein in HeLa cells stimulated the activity of a reporter (pMyc-TA-luc) (compare column 2 with 1), expression of which is driven by the c-Myc-responsive elements. Transfection of a plasmid encoding IFI16 protein resulted in moderate decreases in the activity of the Myc-luc-reporter (compare column 3 with 1). Interestingly, transfection of cells with c-Myc encoding plasmid along with increasing amounts of the plasmid encoding the IFI16 protein significantly reduced the activity of the reporter (compare column 5 or 6 with column 4). Moreover, consistent with our above observations (Fig. 1) that expression of IFI16 protein is inversely correlated with hTERT expression, knockdown of IFI16 expression in HeLa cells resulted in increases in hTERT protein levels (Fig. 3C). These observations indicated that increased levels of IFI16 protein in HeLa cells down-regulate the hTERT expression, in part, through down-regulation of the c-Myc expression and inhibiting c-Myc-stimulated transcription of the target genes.

### Increased Levels of IFI16 Protein in WI-38 Cells Are Associated with Reduced Levels of hTERT and Telomerase Activity

**Figure 4:**
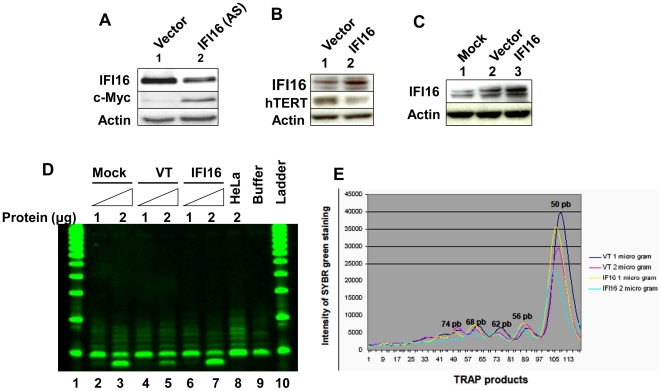
Increased levels of IFI16 protein in WI-38 cells reduce hTERT levels and inhibit telomerase activity. (**A**) Total protein extracts prepared from WI-38 cells transfected with control siRNA (lane 1) or IFI16 siRNA RNA (lane 2) were subjected to immunoblotting using antibodies specific to the indicated proteins. (**B**) Total protein extracts prepared from WI-38 cells, either transfected with control (pCMV) vector (lane 1) or pCMV-IFI16 plasmid (lane 2), were subjected to immunoblotting using antibodies specific to the indicated proteins. (**C**) Total protein extracts prepared from WI-38 cells treated with lipofectamine (mock, lane 1), transfected with control (pCMV) vector (lane 2) or pCMV-IFI16 plasmid (lane 3) were subjected to immunoblotting using antibodies specific to the indicated proteins. (**D**) Extracts containing the indicated amounts (µg) of proteins from mock transfected (lanes 2 and 3)CMV transfected (lanes 4 and 5), or pCMV-IFI16 transfected (lanes 6 and 7) young WI-38 cells were subjected to TRAPeze assays to detect the telomerase activity. As controls, extracts from HeLa cells (lane 8) or buffer alone (lane 9) were also included in the assay. The reaction products were subjected to native polyacrylamide gel electrophoresis along with DNA fragments of increasing lengths as size markers (DNA-ladder, lanes 1 and 10). (**E**) Quantitation (using the Bio-Rad imager) of the intensities of the DNA fragments on the gel (in lanes 4, 5, 6, and 7) that were generated during the TRAPeze assays, which is shown in the panel (**D**).

Our above observations that increased levels of IFI16 protein in HeLa cells reduced c-Myc protein levels (Fig. 3A) and inhibited the c-Myc-stimulated transcription (Fig. 3B), prompted us to determine whether IFI16 protein also regulates the expression of c-Myc and its transcriptional target *hTERT* gene in HDFs. Because c-Myc protein levels were relatively low in WI-38 HDFs [40], we chose to knockdown the expression of IFI16 in young WI-38 HDFs. As shown in Fig. 4A, knockdown of IFI16 expression in WI-38 cells resulted in increases in c-Myc protein levels. Additionally, over-expression of IFI16 protein in WI-38 cells resulted in reduced levels of hTERT protein (Fig. 4B). Similarly, increased levels of IFI16 protein in transfected WI-38 cells (Fig. 4C) also resulted in measurable reduction in the activity of telomerase (Figs. 4D and E; compare lane 7 with either lane 5 or 3). Moreover, consistent with these observations, we also noted that IFN-α treatment of WI-38 cells for 24 h resulted in reduced (∼50%) steady-state levels of *hTERT* mRNA as determined by quantitative real-time PCR (data not shown). Together, these observations indicated that increased levels of IFI16 protein in normal HDFs are associated with reduced steady-state levels of hTERT mRNA and protein and reduced telomerase activity.

### The IFI16 Protein Inhibits c-Myc-induced Transcription of the hTERT Gene

**Figure 5:**
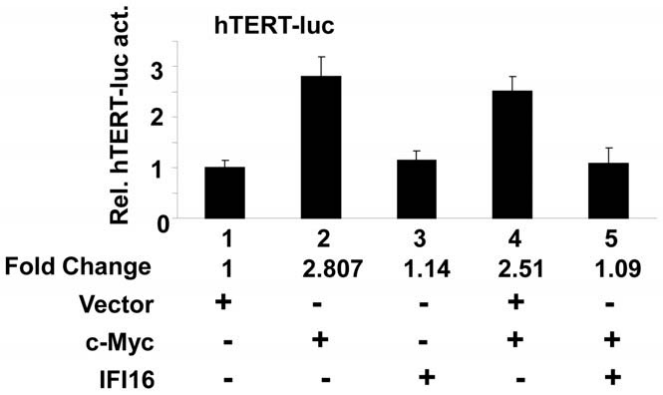
The IFI16 protein inhibits c-Myc-induced transcription of the *hTERT* gene. Sub-confluent cultures of young WI-38 cells were transfected with hTERT-luc reporter plasmid (1.8 µg) along with a second reporter pRL-TK (0.2 µg) plasmid and an empty vector (pCMV), c-Myc encoding plasmid, or IFI16 encoding plasmid as described in methods. 40–44 h after transfections, firefly luciferase and *Renilla* luciferase activities were assayed using dual-luciferase reporter assay kit. Relative luciferase activity was expressed as the ratio of the firefly luciferase and *Renilla* luciferase activity. The numbers indicate fold change in the activity of the firefly luciferase.

c-Myc is known to activate transcription of the *hTERT* gene through *cis*-elements in the promoter region of the gene [39], [40]. Therefore, our above observations that increased levels of the IFI16 protein in cells are associated with reduced expression levels of c-Myc and hTERT mRNA and proteins prompted us to test whether IFI16 protein inhibits c-Myc-stimulated transcription of the *hTERT* gene. For this purpose, we transfected young WI-38 cells with a reporter (hTERT-luc) in which transcription of the reporter was driven by the 5′-regulatory region (∼2 Kb) of the human *hTERT* gene [45]. As shown in Fig. 5, transfection of cells with c-Myc encoding plasmid stimulated the activity of reporter ∼2.5–3-fold (compare column 2 with 1). Consistent with low c-Myc levels in WI-38 cells, transfection of IFI16 encoding plasmid in cells did not result in measurable decreases in the activity of the reporter. However, transfection of IFI16 encoding plasmid along with c-Myc encoding plasmid measurably reduced the activity of the reporter to the basal level (compare column 5 with column 2 or 4). Moreover, we noted that increased expression of c-Myc in WI-38 cells (in transient transfection assays) resulted in increases in hTERT protein levels (data not shown). Together, our observations provided support for the idea that increased levels of IFI16 protein in human normal cells inhibit hTERT expression, in part, by inhibiting c-Myc-stimulated transcription of the *hTERT* gene.

## Discussion

Certain somatic cells are known to express low, but detectable, levels of hTERT [27]–[30] and the levels of hTERT are reported to increase transiently in the S-phase of cell cycle [28], [30]. Therefore, to detect basal low levels of hTERT protein in nuclear extracts prepared from unsynchronized cultures of HDFs, we used our optimized immunoblotting conditions to detect higher molecular weight proteins [47]. Using a mouse monoclonal antibody 2C4, which has been shown to be specific to the human hTERT protein [28], [53] or a specific rabbit polyclonal antibody [54], we could detect the basal low levels of hTERT protein in WI-38 (Fig. 1C) and other young HDFs, such as BJ and IMR-90 (data not shown). Moreover, treatment of cells with aphidicolin increased levels of hTERT mRNA and protein.

Interferon treatment of certain cell types inhibits cell proliferation through inhibition of the progression of cells through the S-phase of the cell cycle [55] and increased levels of IFN-inducible IFI16 protein in HDFs are associated with cellular senescence-associated cell growth arrest [12], Therefore, after detecting the basal levels of hTERT protein in human normal HDFs (WI-38, BJ, and IMR-90), we explored whether IFI16 protein could negatively regulate the expression of hTERT.

Stable overexpression of IFI16 protein in Saos-2 cells resulted in inhibition of cell proliferation and accumulation of senescence-associated β-gal positive cells (a marker of cellular senescence) in cultures [22]. Interestingly, the accumulation of senescent cells was accompanied by up-regulation of p21^CIP1^ expression and down-regulation of c-Myc expression [22]. Consistent with these observations we found that overexpression of IFI16 protein in HeLa cells resulted in decreases in c-Myc protein levels (Fig. 3A) whereas knockdown of IFI16 expression in WI-38 cells resulted in increases in c-Myc protein levels (Fig. 4A). Notably, our observation that knockdown of IFI16 expression in WI-38 cells increased levels of c-Myc is consistent with our previous observations [12] that the knockdown of IFI16 expression increased the proliferation potential of WI-38 cells. Furthermore, overexpression of IFI16 protein in PC-3 human prostate cancer cell line resulted in senescence-like phenotype and reduced telomere length [13], [14]. Together, these observations support the idea that the reduced levels of IFI16 protein in cells contribute to increased proliferation potential through up-regulation of c-Myc and hTERT levels and increased levels of IFI16 protein in cells potentiate cellular senescence-associated cell growth arrest by down-regulating the expression of c-Myc. Because the c-Myc stimulates the transcription of *hTERT* gene [39]–[41], our observations that increased levels of IFI16 protein in cells decrease steady-state levels of c-Myc and inhibit c-Myc-stimulated transcription of the hTERT-luc-reporter support the idea that the IFI16 protein inhibits the transcription of *hTERT* gene by negatively regulating c-Myc-mediated transcription.

**Figure 6:**
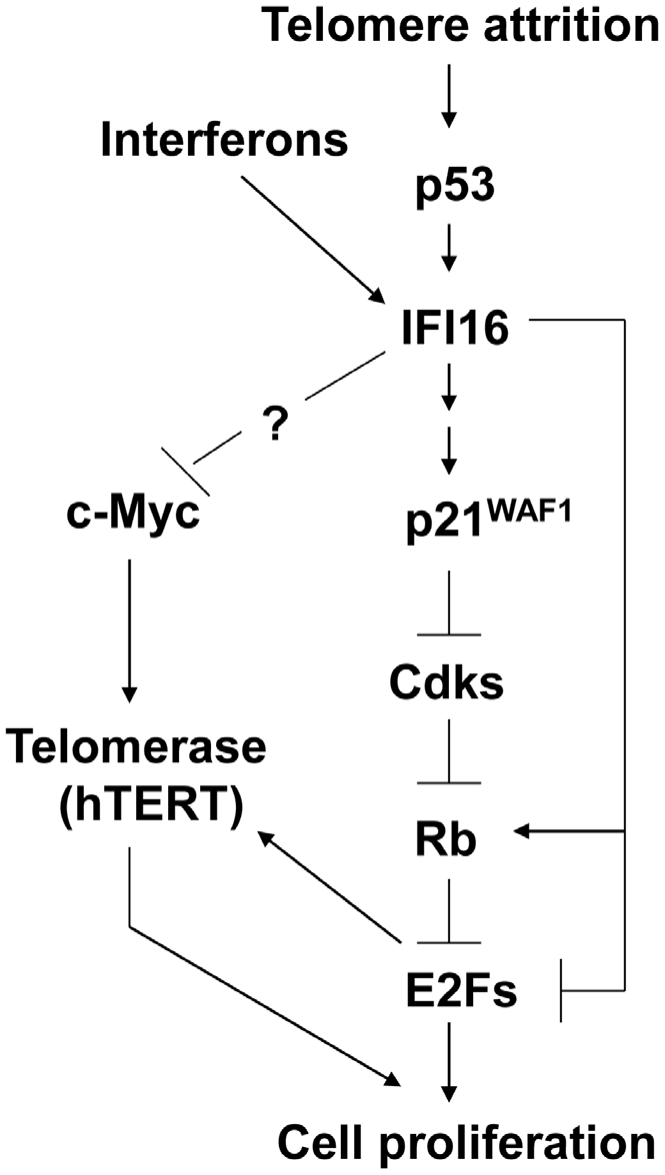
Increased levels of IFI16 protein in human cells inhibit c-Myc-induced expression of the *hTERT* gene.

Presently, it remains unknown how increased levels of IFI16 protein negatively regulate the expression of c-Myc. Because expression of *c-MYC* is regulated through the E2F-binding sites in the promoter region [56], it is tempting to speculate that increased levels of the IFI16 protein in cells inhibit the transcription of *c-MYC* gene by potentiating the pocket protein-E2F-mediated transcriptional repression (Fig. 6). Further work will be needed to test this interesting possibility.

Several studies have suggested that telomere dysfunction and chromosomal aberrations are associated with the aging phenotype associated with Werner syndrome (WS), a rare human premature aging disease that is caused by mutations in the gene encoding the RecQ helicase WRN [57], [58]. Moreover, studies have indicated that telomere elongation by telomerase can significantly reduce the appearance of new chromosomal aberrations in cells lacking the WRN [57], [58]. Because basal and IFN-induced expression of the IFI16 varies among individuals [14], our observations that IFI16 negatively regulates the expression of *hTERT* gene raise the possibility that increased expression of *IFI16* in some individuals, who may have genetic predisposition to have increased serum levels of type I IFNs [59], may contribute to premature aging phenotype. Therefore, further studies are needed to test this possibility.

p53 deficiency is known to rescue the adverse effects of telomere loss and cooperate with telomere dysfunction to accelerate carcinogenesis [60]. Therefore, our previous observations that the p53 transcriptionally activates expression of the *IFI16* gene in old (versus young) HDFs [61] and our current observations that increased levels of IFI16 protein negatively regulate the expression of *hTERT* raise the possibility that the loss of IFI16 expression in human cells (or loss of IFI16 function) by cooperating with telomere dysfunction contributes to carcinogenesis (Fig. 6).

Disruption of telomerase activity in human normal cells slows cell proliferation, restricts cell lifespan, and changes the maintenance of the 3′ single-stranded telomeric overhang without changing the rate of overall telomere shortening [25], [26], [62]. Moreover, telomere dysfunction increases mutation rate and genomic instability [25]. Therefore, it is likely that alterations (increases or decreases) in the expression of IFI16 protein in certain individuals contribute to increased susceptibility to premature aging and aging-dependent cancers.

Previous studies [28], [30] and our observations described here demonstrate that levels of hTERT protein are transiently increased during the S-phase of cell cycle in human normal fibroblasts. Although, the role of telomerase activity in cell cycle progression remains unclear, it is known that hTERT functions independent of the telomerase activity [63]. Therefore, further work will be needed to elucidate the role of hTERT in the S-phase progression.

Inhibitors of HDAC activity, such as TSA and sodium butyrate, are known to inhibit the expression of IFN-inducible genes [49]. Therefore, our observations that treatment of HDFs with CGK1026 resulted in down-regulation of IFI16 expression is consistent with a role for HDAC activity in the regulation of IFI16 expression. Because studies have revealed that dynamic assembly of E2F-pocket protein-HDAC complex plays a central role in the regulation of hTERT expression under normal cycling conditions [29], our observation that treatment of HDFs with CGK1026 resulted in up-regulation of hTERT expression, raises the possibility that increased levels of IFI16 protein in HDFs contribute to transcriptional repression of the *hTERT* gene by the E2F-pocket protein-HDAC repressor complex. Indeed, the IFI16 protein sequence contains two LxCxE-like motifs to bind pRb pocket and it binds to the pRb protein *in vitro* in GST-pull down assays [13]. The IFI16 protein also binds to E2F1 [13]. Binding of IFI16 protein to both pRb and E2F1 is associated with inhibition of the E2F1-mediated transcription of growth-promoting genes whose activity is needed for transit through S-phase of cell cycle [64]. Additionally, over-expression of IFI16 protein in medullary thyroid carcinoma cells significantly down-regulated the expression of the E2F1, a transcriptional repression target of the pRb-E2F repressor complex [65]. Therefore, our observations that treatment of HDFs with CGK1026, which down-regulated the expression of *IFI16*, make it likely that reduced levels of IFI16 protein in cells potentiate the transcription of the E2F1 target genes, such as *hTERT*. Consistent with this idea, we noted that expression of cyclin E and E2F1, well known transcriptional targets of the E2F1 [64], was up-regulated in the CGK1026 treated HDFs (data not shown).

Studies have provided evidence that human epithelial cells and fibroblasts can be immortalized and transformed by over-expression of hTERT, SV40 large T antigen, and activated H-Ras oncogene [66]. Notably, we have reported [12] that immortalization of human fibroblasts with hTERT or SV40 large T antigen down-regulated the expression of the *IFI16* gene and expression of activated H-Ras in human keratinocytes, which resulted in cellular senescence, was associated with up-regulation of IFI16 expression (data not shown). These observations further provide support for the idea that the genetic and/or epigenetic alterations in the human genome that result in defects in cell signaling pathways and immortalization of cells negatively regulate the expression of the *IFI16* gene. Additionally, our observations also raise the possibility that defects in signaling pathways that result in increased expression of the *IFI16* gene in certain individuals contributes to premature ageing and ageing-dependent diseases.
